# Nuclear Control of the Inflammatory Response in Mammals by Peroxisome Proliferator-Activated Receptors

**DOI:** 10.1155/2013/613864

**Published:** 2013-03-07

**Authors:** Stéphane Mandard, David Patsouris

**Affiliations:** ^1^Centre de Recherche INSERM-UMR866 “Lipides, Nutrition, Cancer” Faculté de Médecine, Université de Bourgogne 7, Boulevard Jeanne d'Arc, 21079 Dijon Cedex, France; ^2^Laboratoire CarMeN, UMR INSERM U1060/INRA 1235, Université Lyon 1, Faculté de Médecine Lyon Sud, 165 Chemin du Grand Revoyet, 69921 Oullins, France; ^3^Department of Chemical Physiology, The Scripps Research Institute, MB-24, 10550 North Torrey Pines Road, La Jolla, CA 92037, USA

## Abstract

Peroxisome proliferator-activated receptors (PPARs) are ligand-activated transcription factors that play pivotal roles in the regulation of a very large number of biological processes including inflammation. Using specific examples, this paper focuses on the interplay between PPARs and innate immunity/inflammation and, when possible, compares it among species. We focus on recent discoveries establishing how inflammation and PPARs interact in the context of obesity-induced inflammation and type 2 diabetes, mostly in mouse and humans. We illustrate that PPAR**γ** ability to alleviate obesity-associated inflammation raises an interesting pharmacologic potential. In the light of recent findings, the protective role of PPAR**α** and PPAR**β**/**δ** against the hepatic inflammatory response is also addressed. While PPARs agonists are well-established agents that can treat numerous inflammatory issues in rodents and humans, surprisingly very little has been described in other species. We therefore also review the implication of PPARs in inflammatory bowel disease; acute-phase response; and central, cardiac, and endothelial inflammation and compare it along different species (mainly mouse, rat, human, and pig). In the light of the data available in the literature, there is no doubt that more studies concerning the impact of PPAR ligands in livestock should be undertaken because it may finally raise unconsidered health and sanitary benefits.

## 1. Introduction

The peroxisome proliferator-activated receptors (PPARs) are ligand-activated transcription factors that play critical roles in very different biological pathways such as lipid, protein, glycerol, urea, glucose, glycogen and lipoprotein metabolism, adipogenesis, trophoblast differentiation, and cell migration [1–6]. Notably, PPARs are also required to balance cell proliferation and cell death and therefore impact skin wound healing and proliferative diseases such as cancer [7–9]. PPARs are also prominent players in inflammation control [10, 11]. PPAR*α*, the first PPAR isotype identified in mouse, was originally cloned in the early 1990s as a novel member of the steroid hormone receptor superfamily [12]. Shortly after, a rat version of PPAR*α* as well as three novel members related to each other (xPPAR*α*, xPPAR**β**, and xPPAR**γ**) and to mouse PPAR*α* have been subsequently cloned from *Xenopus* (frog) [13]. Since then, substantial efforts have been made to identify other related receptors; several additional PPAR isoforms and variants have been therefore isolated in a wide range of species including mammals (human, rabbit, mouse, rat, pig, rhesus and cynomolgus monkey, dog, guinea pig, hibernating ground squirrel, and hamster), fishes (grass carp, cobia not only but also marine fish such as the teleost red sea bream (*Pagrus major*) and the mullet *Chelon labrosus*), marine gastropod mollusks (*Cyclostoma*), reptiles (leopard gecko, crocodile, and turtle), and birds (domestic chicken, goose) [14–51].

Since PPARs are ligand-activated transcription factors, a large part of our knowledge about their biological importance is coupled to the function of their target genes. At the molecular level, it was shown that PPARs readily heterodimerize with the Retinoid X Receptor (RXR) prior to ligand binding [52]. In all species tested so far, *Ppar*α**, *Ppar*β*/*δ**, and *Ppar*γ** show specific time- and tissue-dependent patterns of expression (Table 1).

After ligand treatment, the PPAR/RXR heterodimer stably binds on genomic DNA at specific sites called Peroxisome Proliferator Response Element (PPRE) and upregulates gene transcription. Consensus PPREs are formed by two hexameric core binding motifs (AGGTCA) in a direct repeat orientation with an optimal spacing of one nucleotide (DR1). Molecular investigations have demonstrated that PPAR occupies the 5′ motif of the DR1 [53]. Recent analyses have further revealed that even if DR1 PPREs can be located within the promoter sequences of target genes, about 50% of all target sites are located within genes (introns, exons) as well as in 3′ downstream sequences of the target genes [4, 7, 54–58]. The PPAR**α** (NR1C1), PPAR*β*/*δ* (NR1C2), and PPAR**γ** (NR1C3) genes encode proteins that share a highly conserved structure and molecular mode of action, yet the array of genes regulated by each PPAR isotype is divergent and may also differ from one species to another [59]. An extended analysis of the cross-species (mouse to human) conservation of PPREs brought support to this hypothesis because it revealed only limited conservation of PPRE patterns [60]. Strengthening this observation, only a minor overlap between the Wy14,643 (Wy: a specific PPAR**α** agonist) regulated genes from mouse and human primary hepatocytes was found by Rakhshandehroo et al. demonstrating that some, but not all, genes are equally regulated by PPAR**α** in mouse and human hepatocytes [61]. In this review, we explore and focus on the role of PPARs in the control of chronic (mediated by obesity) or acute (as a result of bacterial infection) inflammation in different species, mainly from human, mouse, rat, pig, and cow.

## 2. PPARs and Obesity-Induced Inflammation: Interplay with Adipose Tissue Macrophages 

### 2.1. PPAR*α*

In spite of the relative weak expression level of *Ppar*α** in white adipose tissue (WAT, mainly in adipocytes and not in stromal-vascular cells), several lines of evidence support the notion that PPAR**α** and PPAR**α** agonists could play a functional role in the control of obesity-induced chronic inflammatory response *in vivo*. For instance, treatment of obese diabetic KKAy mice with Wy decreased the mRNA levels of *Tnf-*α** (tumor necrosis factor-**α**), *Mcp-1* (monocyte chemotactic protein-1, also referred to as chemokine (C-C motif) ligand 2, *CCL2*), and *Mac-1* (macrophage antigen-1, also known as cluster of differentiation molecule-11b, *Cd11b*) in epididymal fat, suggesting a reduction in macrophage infiltration [62]. In addition, expression of inflammatory genes in adipose tissue such as *Tnf-*α**, *Mcp-1*, and *IL-1*β** (Interleukin-1 beta) as well as that of specific macrophage markers such as *Cd68 *(macrophage antigen *Cd68*, also known as scavenger receptor class D member 1, *Scard1*),* F4/80 *(also referred to as lymphocyte antigen-71,* Ly71*), and* Adam8* (ADAM metallopeptidase domain 8, also known as cluster of differentiation molecule-156, *Cd156*) in the stromal vascular fraction was more pronounced in *Ppar*α**-deficient mice compared to WT (wild-type) mice rendered obese with a high-fat feeding, reinforcing the notion that PPAR**α** is required for the control of the adipose inflammation process [63]. Another study has also examined the effects of fibrates on the inflammatory changes induced by the interaction between adipocytes and macrophages in obese adipose tissue. Systemic administration of Wy or fenofibrate to genetically obese *ob/ob* mice significantly reduced *Tnf-*α** and *Mcp-1 *mRNA expression in WAT [64]. Similar observation was also reported using adipose tissue explants from *ob/ob* mice suggesting a direct effect of PPAR**α** agonists. To check for the definitive involvement of PPAR**α** in the effects of Wy-mediated reduction in the production of proinflammatory cytokines by white fat pads, adipose tissue explants obtained from PPAR**α**-deficient mice were also used [64]. Compared to WT mice, induction of *Mcp-1* mRNA expression by TNF-**α** (a major paracrine mediator of inflammation in adipocyte) was much robust in adipose tissue explants from *Ppar*α**-deficient mice, suggesting that PPAR**α** is constitutively required to control the steady-state level of adipose *Mcp-1* mRNA levels. Intriguingly, induction of adipose *Mcp-1 *mRNA expression by TNF-**α** was also suppressed by Wy in explants from *Ppar*α**-deficient mice, suggesting that Wy can act independently of the presence of the receptor in fat, at least for the control of the inflammation process [64]. Because *Ppar*γ** is expressed in both mature adipocytes and macrophages, we cannot rule out that part of the effects of fibrates on adipose inflammation are mediated through this other PPAR isotype. Moreover, treating 3T3-L1 mouse adipocytes with Wy or fenofibrate suppressed bacterial lipopolysaccharides-(LPS-) mediated increased in *Mcp-1* mRNA levels, indicating a cell autonomous effect [62]. Interestingly, pharmacological activation of PPAR**α**  also reduced LPS-mediated induction of *Mcp-1* mRNA level in peritoneal macrophages. Therefore, it is possible that PPAR**α** agonists mediate reduction of the inflammatory response in both adipocytes and infiltrated macrophages in WAT. Whether adipose PPAR**α** is a critical factor for the control of adipose inflammation remains a matter for further study. To close this gap, it could be interesting in the future to check for the consequence of the selective deletion of *Ppar*α** in WAT, using the Cre/loxP strategy and the adipocyte/macrophage-specific aP2 (a-FABP) promoter [65].

### 2.2. PPAR*β*/*δ*

While ubiquitously expressed, probably in all cells found in WAT, PPAR*β*/*δ* is also the isotype whose exact roles in the control of WAT function and type-2 diabetes in general are the least clear. Firstly, PPAR*β*/*δ* undoubtedly displays anti-inflammatory properties in numerous cell types present in WAT, such as macrophages, adipocytes, and endothelial cells [66]. In agreement, it was found that activation of PPAR*β*/*δ* prevents LPS-induced NF-*κ*B (a key regulatory proinflammatory transcription factor) activation by regulating ERK1/2 (Extracellular signal-Regulated Kinases) phosphorylation in adipocytes and WAT in mice [67]. PPAR*β*/*δ* may therefore represent an interesting target for the treatment of inflammatory diseases such as atherosclerosis [68]. Secondly, several investigations aiming to determine the role of PPAR*β*/*δ* in WAT mass have demonstrated that it probably only plays a moderate role in adipogenesis and an indirect role in the control of WAT mass [69–72]. For instance, feeding murine models of obesity and diabetes with a PPAR*β*/*δ* agonist decreases their adiposity [73]. Yet, these effects are most likely mediated by *Ppar*β*/*δ** expression in other nonadipose tissues such as liver and skeletal muscle because WAT *Ppar*β*/*δ** conditional knockout mice do not exhibit any apparent adipose tissue phenotype [70]. Furthermore, this indirect role of PPAR*β*/*δ* is also provided in mice overexpressing *Ppar*β*/*δ** in skeletal muscle because these mice display decreased adiposity and adipocyte size [74]. Regarding WAT inflammation, several publications have led to discrepant findings as well. For instance, reconstitution with *Ppar*β*/*δ** null bone marrow of irradiated WT mice to generate *Ppar*β*/*δ**  null animals lacking *Ppar*β*/*δ**  in hematopoietic cells had no clear effects on WAT inflammation and insulin sensitivity. If any benefits on insulin sensitivity were seen, these were different according to the genetic background of the mice and likely mediated by the liver where PPAR*β*/*δ* switches the phenotype of Kupffer cells (liver macrophages-like cells) into an anti-inflammatory phenotype (also called M2 phenotype; this phenotype is acquired after cell activation by cytokines such as Interleukin-4 and Interleukin-13) [66, 75]. Classically, activated macrophages (also known as M1 type) express high levels of proinflammatory mediators that elevate inflammation to a low, but chronic, grade and contribute to insulin resistance [76, 77]. In contrast, M2 “alternatively” activated macrophages are characterized by low production of proinflammatory cytokines (including IL-1**β**, TNF-**α**, and IL-6) and high production of anti-inflammatory cytokines (including IL-10), by a gene expression profile distinct from other macrophage populations and by their capacity to scavenge debris, to promote angiogenesis, tissue repair, and remodeling [78]. However, the observations evoked above contrast with that of Kang et al. who describe that PPAR*β*/*δ* is required for the polarization of adipose tissue macrophages (ATMs) into an M2 phenotype [79]. In summary, the exact role of PPAR*β*/*δ* in the control of WAT inflammation requires further investigations.

### 2.3. PPAR*γ*

In response to an inappropriate diet, insulin resistance settles in WAT further limiting its capacity to store fat. Consequently, excess fatty acids overflow into other organs such as skeletal muscle and liver (ectopic fat), which in turn alters proper functioning of these tissues [80]. PPAR**γ** is strongly associated with obesity because it is highly expressed in white fat depots and it serves as a target for certain anti-diabetic drugs. A substantial amount of *Ppar*γ*1* mRNA level is detected in many tissues including white and brown adipose tissue, skeletal muscle, liver, colon, bone, and placenta and cell types such as pancreatic *β*-cells and macrophages in different species ranging from humans to rodents, sheep and cattle [81]. The other *Ppar*γ**isoform, *Ppar*γ*2*, is highly expressed in WAT in rodents (mainly rats and mice) as well as in humans, chicken, and sheep [20, 82–86].

**Figure 1:**
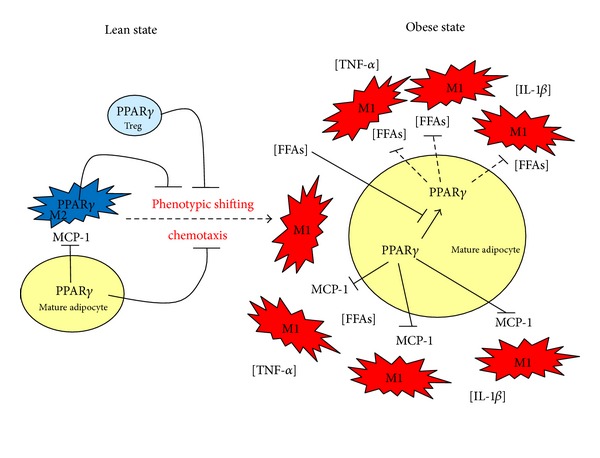
Contribution of the anti-inflammatory roles of PPAR**γ** in the onset of WAT inflammation in the context of obesity and insulin resistance. In the lean state, PPAR**γ** activity maintains homeostasis in mature adipocytes in preventing the secretion of chemokines such as MCP-1. In addition, alternatively activated macrophages (M2) and Treg cells are resident leukocytes in WAT coordinating numerous biological activities such as stimulating angiogenesis and cleaning of dead cells. The role of PPAR**γ** in these cells is to prevent classical activation of macrophages and local inflammation to develop. When obesity is reached, mature adipocytes are exposed to excessive concentrations of free fatty acids (FFAs), which decrease *Ppar*γ** expression. In consequence, insulin sensitivity is also decreased in adipocytes, which elevates even more local FFAs concentrations as adipocytes are no longer able to properly store fatty acids and lipolysis also becomes activated. Furthermore, these FFAs activate macrophages shifting into an M1 phenotype, promoting the release of proinflammatory cytokines such as TNF-**α** and IL-1**β**. Secondly, as PPAR*γ* transrepressional activity is decreased, adipocytes secrete high concentrations of chemokines (MCP-1), further promoting the recruitment of macrophages. Occurrence of this feed forward amplification loop between adipocytes and macrophages eventually leads to the elevation of local inflammation, further exacerbating local insulin resistance, which will turn systemic in the long term. MCP-1: monocyte chemoattractant protein-1; treg cells: regulatory T cells; FFA: free fatty acids; PPAR*γ*: peroxisome proliferator-activated receptor **γ**; TNF-**α**: tumor necrosis factor-alpha; IL-1**β**: Interleukin-1 beta.

A wealth of studies has established the critical role of PPAR*γ* in adipose tissue biology and it is now widely accepted that PPAR*γ* is a predominant nuclear receptor regulating the process of adipose differentiation both *in vivo* and *in vitro* [87–89]. However, it now appears that it is more specifically the low-grade systemic inflammation associated with obesity that is central to the etiology of the disease. During development of obesity, the expansion of WAT is accompanied with increased infiltration of macrophages that accumulate around stressed mature adipocytes [90]. Several genetic and pharmacological manipulations have further revealed situations in which obesity and inflammation were disconnected, demonstrating that obesity as such does not necessarily leads to type-2 diabetes as long as inflammation does not occur [77, 91–93]. In the context of obesity, adipocytes are exposed to excessive concentrations of free fatty acids. We and others have recently demonstrated that various fatty acids, especially arachidonic acid, induce the murine adipose transcription and secretion of chemokines such as MCP-1, Regulated upon Activation, Normal T-cell Expressed and Secreted/chemokine (C-C motif) ligand 5 (RANTES/CCL5), and the chemokine Keratinocyte Chemoattractant (KC, also known as CXCL1) [94–96]. As chemokines govern the recruitment of leukocytes such as macrophages, high-fat diets providing elevated levels of fatty acids are likely to cause the adipose secretion of chemokines. In turn, these chemokines will induce the recruitment of macrophages in WAT and elevate local inflammation (Figure 1). 

Detailed analysis of the molecular mechanisms involved revealed that the activation of the Toll-like receptor 4 pathway (TLR4) by the fatty acids was required. Surprisingly, activation of this pathway causes the decreased expression level of *Ppar*γ**, which was prevented by the cotreatment with ER stress inhibitors [94]. This observation adds up to other publications demonstrating the key, yet unstable, role played by this specialized organelle in maintaining an adequate cellular response to metabolic stresses [97, 98]. Together, this led us to establish a model in which fatty acids, through a TLR4/ER stress-dependent pathway, induce the recruitment of leukocytes by increasing the secretion of chemokines [99].

In spite of decreased *Ppar*γ** mRNA levels, pharmacological activation of PPAR**γ** with rosiglitazone (RSG), a thiazolidinedione (TZD)/PPAR*γ* agonist, prevents fatty acid-mediated adipose induction of chemokines expression and secretion [94, 100]. These observations were strengthened by *in vivo* experiments where treatments of mice fed a high-fat diet by RSG increased adiposity but decreased the expression of chemokines by adipocytes, the classically activated adipose tissue macrophages (M1 type) content and WAT inflammation [77, 94, 101]. Therefore, PPAR**γ** maintains the expression of chemokines to a minimal level in adipocytes. As a member of the nuclear hormone receptor superfamily, PPAR**γ** displays both transactivational and transrepressional activities [59, 102]. Interestingly, it is likely through transrepressional activity that PPAR**γ** affects chemokines secretion by adipocytes [94]. In line with this, it is worth mentioning the recent discovery of MBX-102/JNJ39659100, a member of a novel non-TZD class of selective partial PPAR**γ** agonist with weak transactivational activity, yet high transrepressional activity for PPAR**γ**, that conserves insulin-sensitizing properties without inducing well-known major side effect [103]. As PPAR**γ** transrepressional activity is involved in the repression of proinflammatory cytokines and chemokines, it is tempting to think that part of TZDs therapeutic properties on type-2 diabetes could be explained by their anti-inflammatory properties. Therefore, developing agents able to disconnect the transactivational activity of PPAR**γ** from its transrepressional activity may represent an effective strategy to treat different inflammatory diseases such as type-2 diabetes. This hypothesis raises the fundamental question about how does PPAR**γ** transrepressional activity work? Elucidation of the basic mechanism on how PPAR**γ** controls inflammation has derived primarily from work performed in macrophages [104–106]. As PPAR**γ** transrepressional activity is also involved in the repression of proinflammatory cytokines in the stromal vascular cells of WAT (i.e., the macrophage containing cellular fraction), similar molecular mechanisms of regulation may also occur in adipocytes and macrophages, but it is a nonproven hypothesis at the moment. The scenario is probably as follows: in resting situation, constant binding of corepressors complexes such as nuclear receptor corepressor (NCOR) and silencing mediator for retinoid and thyroid hormone receptors (SMRT) on the gene promoter sequence of these cytokines and chemokines prevent their expression [106]. When an inflammatory stimulus is applied, NCOR becomes ubiquitinated further excluding these complexes from the nucleus. In addition, coactivators are recruited to the promoter of cytokines and transcription of the gene occurs. However, when activated by an agonist of the TZD family, PPAR**γ** becomes SUMOylated and docked to the corepressor complexes [107, 108]. Association between PPAR**γ** and NCOR prevents its ubiquitination further maintaining the expression of chemokines and cytokines in a repressed state. The contribution of PPARs in disconnecting obesity and inflammation is illustrated in genetic models where PPAR isotypes were selectively invalidated in macrophages and bone marrow-derived cells. First, when *Ppar*γ** is invalidated in macrophages, mice become more susceptible to develop insulin resistance, a state that is accompanied with elevated local inflammation in liver, adipose, and skeletal muscle tissues [109, 110]. All the above observations were explained by the shift of macrophages into a proinflammatory (M1 type) phenotype [110]. In consequence, one major role of PPAR**γ** in macrophages is to maintain this population in an alternative anti-inflammatory state (M2 type) expressing genes such as the anti-inflammatory cytokine Interleukin-10, the IL-1 receptor antagonist (IL1-Ra), and arginase I [111, 112].

Another mechanism by which PPAR**γ** controls adipose tissue macrophage polarization in coordinating the metabolism of macrophages. Indeed, classical (M1) activation of macrophages is a highly energy demanding state, which is sustained by glycolytic activity. Alternative (M2) activation of macrophages is less energy demanding and represents a state in which energy supplies are provided by oxidation of fatty acids and glucose. Interestingly, Odegaard and Chawla demonstrated that PPAR**γ** is required to coordinate the oxidative genetic program in macrophages [113]. In support of this notion, it was also demonstrated that the expression of *Ppar*γ** in macrophages is under the control of the pro-M2 cytokine Interleukin-4, which further involves the activation of STAT6 (signal transducer and activator of transcription 6). Finally, PPAR**γ** requires the transcriptional coactivator PGC-1**β** (peroxisome proliferator-activated receptor-gamma coactivator-1) in order to induce the oxidative program supporting macrophages alternative activation. Altogether, this series of observations illustrates that macrophage polarization involves different metabolic pathways that are necessary to sustain their energetic demand, and that PPAR**γ** is coordinating this metabolic activity [113, 114]. 

Besides macrophages, invasion of WAT by neutrophils, eosinophils, B cells, T cells, and mast cells has been also reported. Recently, a small subset of T lymphocytes, the CD4 (+) Foxp3 (+) T regulatory (Treg), were abundantly found in the WAT of normal (lean) but not in different mouse models of obesity [115]. Interestingly, elegant studies have demonstrated that Treg cell depletion in the abdominal adipose tissue led to the induction of proinflammatory transcripts and enhanced inflammatory state of murine WAT [115]. Very recently, Cipolletta et al. found that deleting mouse *Ppar*γ** in Treg cells markedly influences the number of Treg cells residing specifically in WAT and pioglitazone, a synthetic/TZD agonist of PPAR**γ**, and increases substantially the WAT Treg cell population in WT obese animals fed a high-fat diet [116, 117]. Furthermore, the ability of TZDs to downregulate the inflammatory state of WAT and to improve insulin sensitivity was impaired in specific *Ppar*γ**-deficient Treg cells. In conclusion, this information indicates that regulatory T cells expressing *Ppar*γ** are engaged in suppressing adipose tissue inflammation in obesity. Furthermore, PPAR**γ** not only plays an important role in adipose macrophages but also in Treg cells. Further studies are required in order to test whether PPAR**γ** may play a role in other immune cells controlling adipose tissue inflammation and whether this finding can be translated in other species such as humans.

## 3. PPARs and Inflammation in Liver

### 3.1. PPAR*α*

In rodents, *Ppar*α** is abundantly expressed in liver where it regulates a whole array of genes involved in the uptake, binding and degradation of fatty acids by mitochondrial and peroxisomal *β*-oxidation, as well as in lipoprotein assembly, transport and inflammation [118, 119]. More than a decade ago, as PPAR*α* is the nuclear receptor for the eicosanoid leukotriene B4 but also for the palmitoylethanolamide (the naturally occurring amide of palmitic acid and ethanolamine), a role for this nuclear receptor in modulating inflammation was evoked [11, 120, 121]. Since then, a solid body of evidence has implicated PPAR**α** in the duration of inflammation control because prolonged inflammatory response was observed in mice lacking *Ppar*α**, suggesting anti-inflammatory actions for this nuclear receptor [11, 122]. 

#### 3.1.1. Role of PPAR*α* in the Control of Obesity-Induced Inflammation in Liver

The role of PPAR**α** in inflammation has also been studied in the context of obesity-induced chronic low-grade inflammation, which is characterized by increased circulating inflammatory cytokines and acute-phase proteins [123, 124]. Elegant experiments with Sv129 mice lacking the nuclear receptor *Ppar*α** and rendered obese by chronic high-fat feeding displayed an increased abundance of macrophages in liver [63]. In agreement with this observation, mRNA levels of proinflammatory genes were markedly increased in *Ppar*α**-deficient mice fed high fat diet. Because PPAR*α* is a master regulator of fatty acid **β**-oxidation, PPAR**α** may indirectly inhibit inflammation by preventing fat accumulation in liver. However, treatment of mice under nonsteatotic conditions with Wy supports the notion that PPAR**α** is able to downregulate expression of inflammatory genes in liver independently of its effect on hepatic lipid storage [63]. Hence, by reducing hepatic lipid storage (and therefore lipotoxicity) and by suppressing proinflammatory gene expression in liver, PPAR*α* may protect mice from steatohepatitis. These findings were further strengthened by the work of Lalloyer and collaborators who studied the impact of Ppar*α* deletion in apoE2-KI mice (a human like hyperlipidemic mouse model) that were subjected to a Western diet supplemented or not with fenofibrate [125]. These ApoE2-KI *Ppar*α**-knockout (−/−) mice displayed exaggerated liver steatosis and inflammation. Notably, reduced expression of inflammatory markers and macrophage content was observed in WT mice fed fenofibrate but not in *Ppar*α**-knockout mice, highlighting the functional role of PPAR**α** in hepatic inflammation control. Because fenofibrate treatment immediately reduced the expression of inflammatory genes, it was proposed that the beneficial effect of fenofibrate on hepatic lipid disorders (nonalcoholic steatohepatitis) could partly be due to its inhibitory effect on proinflammatory genes [126].

Inasmuch as PPAR**α** is a critical regulator of the hepatic inflammation process, the understanding of how *Ppar*α** expression in the hepatocyte is regulated could provide substantial clues to fight inflammation. In mice, liver *Ppar*α** expression and PPAR*α* activity are strongly reduced by IL-1*β*, a cytokine produced by Kupffer cells, the resident macrophages of the liver [127]. From a molecular point of view, the inhibitory effect of IL-1**β** on *Ppar*α** promoter activity is mediated by the binding of NF-*κ*B to two NF-*κ*B binding sites located in the promoter of the *Ppar*α** gene. Noteworthy, similar molecular mechanism is also observable with the human version of the *PPAR*α** promoter, suggesting possible translation to the human situation. Therefore, strategies aiming at reducing Kupffer cell-derived IL-1**β** could theoretically limit the expansion of inflammation, at least in liver. 

#### 3.1.2. PPAR**α** and the Control of Inflammatory Gene Expression by Transrepression

In addition to upregulation of gene expression, a growing body of evidence in the scientific literature indicates that PPAR**α** also displays significant transrepressional activities on inflammatory genes. In agreement, PPAR**α** has been shown to interfere with several proinflammatory transcription factors including STAT, activator protein-1 (AP-1), nuclear factor-kappa B (NF-*κ*B), and nuclear factor of activated T cells (NFAT). NF-*κ*B activity is tightly controlled by the degradation of the inhibitory protein I*κ*B-alpha (I*κ*B*α*) that retains NF-*κ*B dimers in a nonactive form in the cytoplasm. It is worth recalling that PPAR**α** upregulates the expression of I*κ*B**α** in human aortic smooth muscle cells as well as in primary human hepatocytes [128]. Upon activation of I*κ*B*α*, the nuclear translocation and DNA-binding activity of the proinflammatory transcription factor NF-*κ*B is suppressed. Induction of I*κ*B*α* expression can be seen as one of the mechanisms that contribute to the anti-inflammatory activities of PPAR*α* activators. It was also reported that pharmacologically activated PPAR**α** was capable to sequestrate the coactivator glucocorticoid receptor-interacting protein-1/transcriptional intermediary factor-2 (GRIP1/TIF2), leading to a reduced activity of the proinflammatory transcription factor CAATT/enhancer binding proteins (C/EBP) that ultimately cannot anymore transactivate the fibrinogen-**β** gene in liver [129]. By virtue of their anti-inflammatory abilities, glucocorticoids are among the most commonly prescribed medications for the treatment of acute and chronic inflammatory diseases. Simultaneous activation of PPAR**α** and glucocorticoid receptor alpha (GR**α**) enhances transrepression of NF-*κ*B-driven gene expression and additively represses proinflammatory cytokine production [130]. This finding paves the road for new approaches for the treatment of inflammatory diseases where the additive effect of PPAR**α** and GR**α** activation could repress to a larger extent the inflammatory gene expression program.

#### 3.1.3. Direct Upregulation of Anti-Inflammatory Genes by PPAR*α*

PPAR**α** has been first described as a ligand-activated transcription factor across species and as such it directly upregulates a certain array of genes. In addition to downregulating expression of proinflammatory genes, PPAR**α** could therefore theoretically suppress the inflammatory response by direct upregulation of gene(s) with anti-inflammatory properties. Surprisingly, only a very limited number of inflammatory genes have been identified so far as direct PPAR**α** positive targets. Searching for novel direct PPAR**α** reguled genes in liver, we previously identified the Interleukin-1 receptor antagonist (IL1-Ra) gene as an additional mechanism for PPAR**α** to negatively regulate the APR in mouse liver [131]. It is noteworthy that upregulation of *IL-1ra* by PPAR**α** was conserved in human (HepG2 hepatoma cells and human monocyte/macrophage THP-1 cell line) supporting the notion that similar regulation likely occurs in humans [131, 132]. Furthermore, using mice deficient in *Ppar*α** combined with pharmacological activation of PPAR**α** by the synthetic PPAR*α* agonists Wy, fenofibrate, or clofibrate, two different groups found that the liver expression of *Vanin-1* (a glycosylphosphatidylinositol-linked membrane-associated pantetheinase that promotes the production of inflammatory mediators by intestinal epithelial cells) was directly regulated by PPAR**α** in mice [118, 133]. Treatment of primary human hepatocytes or HepaRG cells (a cell line derived from a liver tumor of a female patient) with two different PPAR**γ** agonists (RSG and troglitazone) also modulate the mRNA levels of *Vanin-1* indicating that similar to PPAR**α**, *Vanin-1* could be regulated by PPAR**γ** [134]. *In vivo* upregulation of *Vanin-1* in the liver of mice by the di(2-ethylhexyl) phthalate (DEHP), a synthetic PPAR**γ** ligand, has been also reported [135]. The question arises, why an anti-inflammatory transcription factor such as PPAR**α** would increase the expression of *Vanin-1* that rather promotes the inflammation process. At present, it is hard to reconcile the Wy-mediated upregulation of *Vanin-1* mRNA level in liver with the anti-inflammatory role of PPAR**α**. Follow-up investigations are eagerly awaited to partly close this gap. Additionally, the group of S. Kersten also reported on the direct and critical role of human PPAR**α** in the hepatic regulation of the mannose-binding lectin (*MBL*) gene, a soluble mediator of innate immunity [136]. Given that *MBL* is an important player in complement cascade activation as part of the first-line host defense, the positive regulation *MBL* fits within the role of PPAR**α** as important regulator of inflammation and innate immunity. 

### 3.2. Possible Role of PPAR*β*/*δ* in the Control of Inflammation Process in Liver

Similar to PPAR**α**, the nuclear hormone receptor *Ppar*β*/*δ** is expressed in the liver and displays anti-inflammatory activities. For instance, mice fed the PPAR*β*/*δ* agonist L-165041 are partially protected from chronic ethanol-mediated hepatic injury and inflammation [137]. Yet, others have reported that PPAR*β*/*δ* would promote hepatic stellate cell proliferation during acute and chronic liver inflammation, favouring the onset of hepatic tissue injury [138]. Therefore, the role of PPAR*β*/*δ* in liver is not fully understood and it deserves further investigations. In an attempt to define the functional role of PPAR*β*/*δ* in the liver in mice, the group of S. Kersten and collaborators has used Affymetrix microarrays to compare the RNA populations of normally fed wild-type mice *versus *mice deficient in the *Ppar*β*/*δ**isoform [139]. *Ppar*β*/*δ** deletion was associated with enrichment of gene sets involved in various innate immunity and inflammation-related processes including natural killer cell-mediated cytotoxicity, antigen processing and presentation, and Toll-like receptor pathway. Significant higher expression of genes reflecting enhanced nuclear factor-kappa B (NF-*κ*B) activity was found in *Ppar*β*/*δ** null mice [139]. Elevation of Kupffer cell (the resident macrophages in liver) marker gene expression was also observable. Enhanced expression of proinflammatory genes that are regulated by the NF-*κ*B signaling was also noted in *Ppar*β*/*δ** null mice following administration of the prototypical liver-specific toxicant carbon tetrachloride (CCl4) administration [140]. Of interest, normal-diet fed mice infected by adenovirus overexpressing *Ppar*β*/*δ** in liver displayed reduced hepatic proinflammatory cytokines/chemokines (*IL-1*β**, *Tnf-*α**, *Ifn-*γ**(*interferon-*γ**), and *Mcp-1*) gene expression by the activated proinflammatory M1 macrophages [141]. In contrast, markers for the alternative anti-inflammatory M2 macrophage activation such as *Mrc1* (mannose receptor, C type 1, also known as Cluster of differentiation molecule-206, *Cd206*) and *Mgl1* (*galactose-type C-type lectin 1,* also referred to as *Cluster of differentiation molecule-301*, *Cd301*) were upregulated in the liver. Others have also reported that genetic deletion of *Ppar*β*/*δ** in mice impaired the alternative anti-inflammatory M2 activation of hepatic macrophages (Küppfer cells) [75]. It was concluded that PPAR*β*/*δ* transcriptional signaling was required for the maintenance of alternative anti-inflammatory M2 activation of Kupffer cells in liver and for the decreased production of proinflammatory cytokines by the proinflammatory M1 macrophages. Curiously and in agreement with findings from Staels' group, these regulations were lost in mice fed a high-fat diet, casting doubt on the real impact of PPAR*β*/*δ* in decreasing obesity-induced hepatic inflammation in mice [141]. 

Inflammatory processes are generally considered to follow the transition of steatosis (simple fatty liver) to nonalcoholic steatohepatitis (NASH) and are therefore regarded as a characteristic finding of NASH. Intriguingly, it was recently found that the PPAR*β*/*δ* agonist GW0742 could attenuate hepatic steatosis by reducing liver triglyceride content and proinflammatory cytokines liver gene expression on a type-2 diabetic rat model [142]. However, this study did not aim at determining the impact of Kupffer cells on hepatic triglyceride storage and liver tissue inflammation. Consequently, unlike for PPAR**α**, whether GW0742 involves some actions on Küppfer cells to prevent NASH is not documented. 

Supporting further PPAR*β*/*δ*'s anti-inflammatory activity, treatment of mice with GW0742 or KD3010, two PPAR*β*/*δ* agonists, significantly reduced copper-induced proinflammatory and APR cytokines in liver of mice [143, 144]. In contrast, blockade of the PPAR*β*/*δ* signaling pathway by the PPAR*β*/*δ* antagonist GSK0660 reverted copper-induced liver damages. Together, these findings support the notion that pharmacological activation of PPAR*β*/*δ* could become an important tool in the management of liver inflammation. 

#### 3.2.1. Humanized Mice for hPPAR*β*/*δ*: Role in Inflammation Control in Liver

In order to investigate whether the human version of PPAR*β*/*δ* also displays similar anti-inflammatory properties, a mouse model humanized for the PPAR*β*/*δ* isoform (PPAR*β*/*δ* KI) was established in a C57BL/6J-stabilized genetic background [145]. Subsequent experiments have shed light on the role of human PPAR*β*/*δ* on liver inflammation in the context of diet-induced obesity in mice. Similar to PPAR**α**, pharmacological activation of PPAR*β*/*δ* (both of human and mouse origins) by the synthetic GW0742 compound led to the comparable induction of the liver *IL1-Ra* mRNA levels in WT and PPAR*β*/*δ* KI C57BL/6J mice. Moreover, it similarly decreased the gene expression of the proinflammatory cytokine *Tnf-*α** and that of the APR proteins *fibrinogen-*α** and *fibrinogen-*β** [145]. These observations support the notion that the mouse *IL1-Ra* gene is likely transcriptionally regulated by the multiple PPAR isotypes and that PPAR*β*/*δ* plays anti-inflammatory functions in liver.

### 3.3. PPAR*γ*: Role in the Control of Inflammation Process in Liver

A wealth of study has previously established a link between obesity and inflammation in the liver. Notably, excessive neutral lipids (triglycerides) accumulation in the liver can first lead to steatosis that may progress to steatohepatitis and ultimately to cirrhosis. In an effort to selectively study the functional role of liver PPAR**γ** in obesity-induced hepatic inflammation, mice deleted of *Ppar*γ** in hepatocytes using the cell type-specific gene-knockout technology were recently established [146, 147]. While these mutant mice were protected against high-fat diet-induced hepatic steatosis, the number of liver inflammatory foci and the concentration of circulating inflammatory markers such as TNF-**α** and MCP-1 were similar as to control mice. These data argue against a predominant role of the liver form of PPAR**γ** in controlling proinflammatory cytokine gene expression in the context of obesity-induced inflammation. 

Many of the effects of TZDs are independent of PPAR**γ** [148]. Supporting this notion, 15-deoxy-Δ12,14-prostaglandin J2 (15d-PGJ2), a natural PPAR**γ** agonist, was found to reduce the recruitment of bone marrow-derived monocyte/macrophages (BMDM) in the liver of mice suffering from cholestasis-induced hepatic inflammation [149]. The suppression of BMDM migration did not result from the direct activation of PPAR**γ** because the inhibitory effect of 15d-PGJ2 on BMDM migration was not affected by the pharmacological antagonization of PPAR**γ**. Rather, 15d-PGJ2 reduced BMDM migration through ROS formation. Therefore, it should be acknowledged that some of the effects of TZDs on the inflammation process are independent of PPAR**γ**. 

## 4. PPARs and the APR across Species 

The complex series of reactions initiated in response to infection and inflammation, trauma, burns, ischemic necrosis, and malignant tumors is called the APR. It is present in all animal species and constitutes a core component of the innate immune system. These alterations are mostly mediated by proinflammatory cytokines, and if prolonged, they contribute to a variety of ailments such as dyslipidemia, atherogenesis, diabetes, mitochondrial dysfunction, and muscle mass loss. Interconnections between APR and PPARs are illustrated by the reduction of PPAR expression in response to bacterial LPS exposure in numerous tissues such as liver, heart, kidney, and WAT [150–152]. This observation actually extends to most of type II Nuclear Hormone Receptors (NHRs) [153–155]. The prevalently accepted anti-inflammatory role for PPARs suggested that their agonists may be able to counterbalance APR-induced inflammation. In particular, the protective roles of PPARs were evaluated in response to endotoxemia induced by *Escherichia coli* LPS. 

### 4.1. PPAR*α* and the APR

Regarding PPAR**α**, treating mice model of endotoxemia with fenofibrate or Wy surprisingly elevated TNF-**α** levels in plasma [156]. This elevation was not observed in *Ppar*α** knockout mice, further establishing a functional role of PPAR**α** in mediating this effect of LPS [157]. Furthermore, some authors reported that C57BL/6 mice injected intraperitoneally with 100 *μ*g of LPS (*Escherichia coli* LPS, serotype 055:B5) displayed a marked reduction in *Cyp4a10* (cytochrome P450, family 4, subfamily a, polypeptide 10) mRNA levels in the kidney [158]. Intriguingly, LPS-mediated reduction of *Cyp4a10* expression was still observable in the kidneys of *Ppar*α*-*deficient mice. This finding suggests that mouse PPAR**α** does not trigger the effects of LPS on *Cyp4a10* expression in the kidney [158]. Surprisingly, others found that injection of purified LPS (*Escherichia coli* LPS, serotype 0127:B8) in mice was inducing cytochrome *Cyp4a10 and Cyp4a14* (cytochrome P450, family 4, subfamily a, polypeptide 14) expression in kidney, in a PPAR**α**-dependent manner [159]. Downregulation of *Cyp2a5*, *Cyp2c29,* and *Cyp3a11* by LPS was also comparatively reduced in *Ppar*α** null mice, suggesting that PPAR**α** is somehow required for LPS-mediated gene regulation and could serve the purpose of LPS-mediated inflammation [159]. 

A profound role of PPAR**α** in counteracting inflammation during APR is also illustrated by the fact that wild-type C57BL/6 mice injected intraperitoneally with proinflammatory cytokines such as TNF-**α** and IL-1**β** (two potent inducers of APR) display a significant reduction in hepatic mRNA levels of *Ppar*α** and its obligate partner *Rxr*α** [155]. Similar results were also obtained using the human hepatoma Hep3B cell line; these data are in agreement with those reported by Stienstra and colleagues who recently disentangled the molecular mechanisms responsible for this reduction in *Ppar*α** mRNA levels [160]. Notably, further analysis revealed that the DNA binding of the heterodimer PPAR**α**/RXR**α** to cognate peroxisome proliferator-responsive elements was significantly reduced [155]. This interesting piece of data explains, at least partially, why the expression of well-known *Ppar*α**-regulated transcripts is also concomitantly reduced [155]. Thus, by downregulating *Ppar*α** expression and PPAR*α* activity in liver, LPS challenge may limit fatty acid **β**-oxidation. As a consequence, LPS would favor a metabolic shift in fatty acid metabolism by promoting their esterification and accumulation in the liver, ultimately leading to sepsis-induced hypertriglyceridemia.

In humans, it was recently shown that fenofibrate did not perform better than placebo in a cardiometabolic inflammation model where healthy adults were treated with LPS [161]. However, several observations also indicated that PPAR**α** had beneficial effects against endotoxemia in humans. In spite of the relative low hepatic expression of *PPAR*α** in human, its pharmacological activation using fenofibrate or bezafibrate has been shown to decrease plasma levels of several APR proteins that are normally increased during inflammatory conditions [162–164]. Furthermore, PPAR**α** activation by fenofibrate also prevents myocardial dysfunction during endotoxemia in rats [165]. 

Another line of evidence connecting PPAR**α** to the control of inflammation gene expression came with the use of a liver-restricted *Ppar*α** expression mouse model that was treated with bacterial LPS [166]. Using mice deficient in *Ppar*α** in all tissues except the liver, a specific liver action of PPAR**α** was highlighted because the hepatic expression and circulating levels of proinflammatory cytokines were comparatively lower in the mutant animals [166]. These findings support the notion that PPAR**α** readily reduces the stimulation of the acute phase response (APR). 

Hence, while PPAR**α** is likely a factor playing a determinant role in the control of hepatic inflammation, its ability to control APR still deserves to be clearly unraveled.

### 4.2. PPAR*β*/*δ* and the APR

Information on the role of PPAR*β*/*δ* in the pathophysiology of sepsis-induced organ dysfunction and injury still remain fragmentary at the moment. In an effort to better investigate the role of PPAR*β*/*δ* in murine model of LPS-induced sepsis, WT and *Ppar*β*/*δ**-deficient mice-previously subjected to LPS, were given the selective PPAR*β*/*δ* ligand (GW0742). Notably, GW0742 attenuated the degree of LPS-induced pulmonary inflammation, as well as cardiac and renal dysfunction [156, 167]. In further support of a role of PPAR*β*/*δ* in endotoxemia, LPS-treated WT and *Ppar*β*/*δ**-deficient mice were also given GSK0660 (a synthetic PPAR*β*/*δ* antagonist). Interestingly, most of the beneficial effects of GW0742 on the reduction of the septic shock was abolished [167]. PPAR*β*/*δ* may therefore represent an attractive method to counteract APR.

### 4.3. PPAR*γ* and the APR

Similar to PPAR**α**, results obtained on the role of PPAR**γ** led to inconsistent observations, at least in rodents. The protective roles of PPAR**γ** were particularly evaluated in response to endotoxemia induced by *Escherichia coli *LPS. It is worth recalling that RSG-induced activation of PPAR**γ** in rats subjected to *Escherichia coli* LPS challenge alleviates LPS-mediated proinflammatory cytokine production in lungs inflammation models [168, 169]. Other studies performed with male Wistar rats also concluded that the beneficial protection of the 15d-PGJ2 against the multiple organ failure caused by endotoxin was mediated partially through PPAR**γ** [170]. It was proposed that once activated, PPAR**γ** would attenuate LPS-induced release of high mobility group box 1 in blood, a well-known late proinflammatory mediator of sepsis [171]. However, it should be stressed that others found that pharmacological activation of the PPAR**γ** isotype was not useful for the treatment of acute inflammation in lean or *db/db* mice, raising doubts about the routine use of PPAR**γ** agonists as anti-inflammatory agents in clinical applications [172]. The picture is even more complex because treating weaned pigs with RSG has been shown to be effective to protect them from LPS-induced intestinal damage, as the probable consequence of the inhibited production of intestinal proinflammatory mediators [173]. In conflict with these data, activation of PPAR**γ** with RSG did not ameliorate and even worsened proinflammatory cytokine production in weaned pigs after *Escherichia coli *LPS challenge, casting doubts about the prevalently accepted anti-inflammatory role for PPAR**γ** activation [174]. 

## 5. PPARs in Inflammatory Bowel Disease (IBD)

Characterized by an unrelenting destruction of the gut mucosa, the global prevalence rate of IBD is rising steadily. Ulcerative colitis and Crohn's disease are the two major forms of idiopathic IBD. These complex inflammatory diseases are usually developed in the second and third decades of life. Several players are involved in the onset of the disease among which not only different intestinal cells (intestinal epithelial cells, Paneth and goblet cells), second innate (macrophages, dendritic cells), and adaptive immune cells (lymphocytes), but also luminal bacteria. Collectively, scientific publications on IBD have established that the disease appears to involve maladaptive responses of the body to the intestinal flora, which also depends on individual genetic susceptibility. 

Interestingly, all three PPAR isotypes are detected in the gastrointestinal tract. In rodents, *Ppar*α** is highly expressed in the proximal part of the small intestine (duodenum, jejunum) and colon but to a much lesser extent [175–177]. Expression of human *PPAR*α** expression also peaks in the small intestine and is less in the colon [118, 175]. Regarding mouse *Ppar*β*/*δ**, its expression is highest in the epithelial cells of the colon and much less in small intestine [176].

### 5.1. PPAR*α* in IBD

The role of PPAR**α** during colonic inflammation has been well documented in several studies. In a model of IBD in mice, proinflammatory cytokines formation such as TNF-**α** and IL-1**β** was significantly higher in colon samples from *Ppar*α**-deficient mice compared with those of WT mice [178]. Furthermore and as it could be expected, administration of Wy or fenofibrate to mice suffering from colitis decreased mortality as well as mRNA levels of proinflammatory cytokines (*Ifn*γ**, *Tnf-*α**, *IL-6*, *IL-1*β**, and *Interleukin-17*) in the distal colon leading to an overall delay in the onset of the disease [177]. Notably, the Wy lowering degree of colitis is PPAR**α** dependent [179]. Together, these results indicate that PPAR**α** and PPAR**α** ligands may play an important role in controlling colonic inflammation through the activation of PPAR**α**. 

### 5.2. PPAR*β*/*δ* in IBD

Concerning *Ppar*β*/*δ**, its deletion in mice resulted in exacerbated dextran sulfate sodium-induced colitis suggesting that this nuclear receptor could play a functional role against inflammatory colitis [180]. However, pharmacological activation of PPAR*β*/*δ* did not protect against dextran sulfate sodium-induced colitis pointing towards a ligand-independent anti-inflammatory effect of PPAR*β*/*δ*. More studies need to be done in order to clarify its role in the reduction of IBD.

### 5.3. PPAR**γ** in IBD

With respect to *Ppar*γ**, its expression is restricted to the distal part of the intestine, especially caecum and colon [83, 176, 181–184]. Supporting a potential role of PPARs in IBD, colonic epithelial cells from ulcerative colitis patients express considerably lower levels of *PPAR*γ** [185]. In line with a role of PPAR**γ** in the management of IBD, it is worth recalling that natural (such as conjugated linoleic acid) or synthetic PPAR*γ* agonists provide effective treatments of colitis in rodent experimental models of the disease, but whether only PPAR**γ**-dependent mechanisms are involved remains an open issue [186]. Illustrating the close ties between PPAR*γ* and IBD, mice with targeted disruption of the *Ppar*γ** gene in intestinal epithelial cells displayed increased susceptibility to dextran sodium sulfate-induced colitis as well as higher mRNA levels of proinflammatory markers in the colon [187]. 

Notably, physical association of PPAR*γ* with the transcription factor NF-*κ*B (p50-Rel A heterodimer) has also recently emerged as a novel crucial mechanism by which PPAR**γ** could also limit inflammation in epithelial cells of the gut exposed to *Bacteroides thetaiotaomicron*, a chief component of commensal gut microflora and a prevalent anaerobe of the human intestine [188]. The newly formed PPAR*γ*/NF-*κ*B p50-Rel A complex is rapidly exported from the nucleus resulting in the attenuation of NF-*κ*B-mediated inflammation gene expression. Pharmacological modulation of this PPAR*γ*-dependent anti-inflammatory mechanism might be promising for fighting IBD.

Given the critical role of PPAR**γ** in controlling the activity of NF-*κ*B, it is surprising that none of the 22 human PPAR*γ* genetic variants identified and tested by Mwinyi et al. was associated with IBD susceptibility or disease course; in view of these results, the question still comes up, if PPAR**γ** is indeed a true modulating risk factor for IBD in humans [189].

Whereas *Ppar*γ** is abundantly expressed in intestinal epithelial cells, it is also highly expressed in macrophages and T cells. Genetic rodent models where *Ppar*γ** has been specifically invalidated in these cells have clearly indicated that PPAR**γ** has protective effects on IBD [190–192]. Different mechanisms have been proposed so far however PPAR*γ* anti-inflammatory property appears to be central to its benefits. In intestinal epithelial cells, different reports established that the ability of PPAR*γ* to alter TLR2 and TLR4 signaling is an important factor. This is an interesting observation given the role of luminal flora in IBD because TLR2 and TLR4 are receptors sensing microbe components such as LPS of gram-negative bacteria. In addition, goblet and Paneth cells are also implicated in IBD. Whereas Paneth cells have a protective role against Crohn's disease, goblet cells protect against colitis. Whether PPARs have a role in the function of these cells in IBD remains unclear at the moment. 

## 6. PPARs and Central Inflammation

Diseases of the central nervous system (CNS) present a challenge for the development of new therapeutic agents. *Ppar*γ**, *Ppar*α*,* and *Ppar*β*/*δ** isoforms are expressed in the CNS at different levels, with *Ppar*β*/*δ** being the most abundant [86, 193–196]. 

### 6.1. PPAR*α* in Central Inflammation

In the CNS, the expression of *Ppar*α** has been described in brain and spinal cord [193, 196, 197]. To evaluate the possible role for PPAR*α* at the CNS level in mediating peripheral inflammation, the PPAR**α** agonist GW7647 was intracerebroventricularly injected in mice subjected to carrageenan-induced paw edema [198]. Interestingly, specific activation of central PPAR**α** controls inflammation in the spinal cord as well as in the periphery. It was concluded to the existence of a centrally mediated component for the anti-inflammatory effects of PPAR**α** agonists. 

### 6.2. PPAR*β*/*δ* in Central Inflammation

There are several lines of evidence supporting that PPAR*β*/*δ* serves a critical role in central inflammation. For instance, pharmacological activation of PPAR*β*/*δ* in rat aggregating brain cells cultures with the synthetic compound GW501516 decreased IFN**γ**-induced TNF**α** and INOS in a similar manner to what has been reported in isolated cultures [199]. Further supporting anti-inflammatory function for PPAR*β*/*δ*, oral administration of the selective PPAR*β*/*δ* agonist GW0742 in a mouse of experimental autoimmune encephalomyelitis, reduced astroglial and microglial inflammatory activation as well as IL-1**β** levels in brain [200]. Activation of PPAR*β*/*δ* by the gemfibrozil molecule (an FDA-approved lipid-lowering drug) was also recently shown to be beneficial for the correction of bacterial LPS-mediated inflammation in human microglia, suggesting that central PPAR*β*/*δ* could be a novel interesting molecular target [201]. Follow-up studies have thereafter investigated if central PPAR*β*/*δ* could indeed play a role in the control of CNS inflammation. Supporting this hypothesis, it was found that mice with specific deletion of *Ppar*β*/*δ** in hypothalamic neurons exhibited elevated markers of hypothalamic inflammation such as IL-6 and IL-1**β** [202]. Mutant mice fed a high-fat diet were also found to be resistant to further activation of hypothalamic inflammation. Central PPAR*β*/*δ* appeared therefore as a critical transcription factor in the management of CNS inflammation and lipid accumulation [202]. 

### 6.3. PPAR*γ* in Central Inflammation

Over the past few years, PPAR*γ* has been investigated for its action in ameliorating the development and progression of a number of CNS diseases. Because PPAR*γ* agonists exhibit potent anti-inflammatory effects, the hypothesis was raised that they could display direct neuroprotective actions. Animal models of Alzheimer's disease or Parkinson's disease fed pioglitazone, a PPAR**γ** agonist of the TZDs family, indeed displayed reduction in central inflammation and limited progression of the disease [203, 204]. The availability of FDA-approved agonists of this receptor should facilitate the rapid translation of these findings into clinical trials for a number of CNS diseases. 

## 7. PPARs and Cardiac Inflammation

Heart failure patients show elevated plasma levels of proinflammatory cytokines suggesting that chronic inflammation could play an important role in cardiac diseases such as the development of cardiac hypertrophy. Cardiac hypertrophic and inflammatory pathways are intrically connected because they both activate NF-*κ*B. PPARs isoforms are all present in cardiac muscle cells of mice and rats even though the *Ppar*γ** isoform is expressed at relatively low level [205]. 

### 7.1. PPAR**α** in Cardiac Inflammation

Not only is *Ppar*α** highly expressed in liver, it also plays a very important role in cardiac inflammation. One illuminating set of experiments carried out with hypertensive rats, fed or not the PPAR**α** activator fenofibrate, brings support to the notion that PPAR**α** is also capable to decrease expression of inflammatory genes associated with NF-*κ*B [206]. The anti-inflammatory effect of PPAR**α** was further supported by other studies conducted in hearts of WT and *Ppar*α**-deficient mice. Notably, deletion of *Ppar*α** had a marked effect on the expression of genes related to inflammation and immunity [207]. In the context of cardiac hypertrophy (which is characterized by induction of inflammatory pathways), mRNA levels of genes, known to be under the dependence of the transcription factor NF-*κ*B and therefore involved in inflammation and immunity, were decreased in neonatal rat cardiomyocytes treated with Wy or infected with adenoviruses overexpressing *Ppar*α** [208, 209]. Together, these data point to a pivotal role of PPAR**α** in limiting the inflammatory response by transrepression of NF-*κ*B in cardiomyocytes.

### 7.2. PPAR*β*/*δ* in Cardiac Inflammation

Interestingly, adenoviral-mediated overexpression of *Ppar*β*/*δ** in cultured neonatal rat cardiomyocytes substantially inhibited LPS-induced *Tnf*α** expression [210]. In support of this result, pharmacological activation of the PPAR*β*/*δ* isotype with the GW501516 molecule prevented the proinflammatory profile induced by lipids in heart and human cardiac AC16 cells [211]. Global and cardiomyocyte-restricted deletion of *Ppar*β*/*δ** in mice has also definitively been instrumental in identifying PPAR*β*/*δ* as a critical nuclear receptor controlling proinflammatory cytokines production in response to LPS treatment in cardiomyocytes [210, 211]. It was concluded that absence of *Ppar*β*/*δ** in cardiomyocytes further exaggerated LPS and lipid-induced proinflammatory cytokine production in heart. 

### 7.3. PPAR*γ* in Cardiac Inflammation

Besides metabolic effects, activation of PPAR**γ** may also promote anti-inflammatory responses in heart. In agreement with this, mice infected by *Trypanosoma cruzi* (also known as *Schizotrypanum cruzi*) display intense inflammatory infection in cardiomyocytes. Supporting the assertion that PPAR**γ** is a potent modulator of the inflammatory process, its selective activation by the 15d-PGJ2 inhibited the expression and activity of different inflammatory enzymes and proinflammatory cytokines in neonatal mouse *Trypanosoma-cruzi*-infected cardiomyocytes [212, 213].

## 8. PPARs, Inflammation, and Endothelium

### 8.1. PPAR*α* and the Control of Endothelial Inflammation

Pharmacological activation of endogenous PPAR**α** from porcine pulmonary-arterial endothelial cells or from human vascular endothelial cells with selective agonists reduced TNF-**α** –mediated induction of inflammatory transcription factors *NF*-*κB* and *AP-1* and expression of their target genes *Vcam-1* and *IL-6*. This piece of data suggests that irrespective of the species, PPAR**α** is a molecular target that, once activated, reduces the proinflammatory phenotypes in endothelial cells [214, 215].

### 8.2. PPAR*β*/*δ* and the Control of Endothelial Inflammation

While the function of the PPAR*β*/*δ* isotype largely remained an enigma until the last century, probably because of the lack of connection with evident clinical manifestations, knowledge concerning its impact on inflammation in endothelial cell has tremendously increased over the last few years. Supporting this statement, treatment of primary vascular endothelial EAhy926 cells with the Merck ligand PPAR*β*/*δ* activator L-165041 suppressed TNF**α**-induced adhesion molecule (such as VCAM-1 and MCP-1) through significant reduction in the nuclear translocation of NF-*κ*B [216, 217]. Furthermore, treating human umbilical vein endothelial cells (HUVEC) with the same molecule reduced the levels of C-reactive protein-mediated increase of Interleukin-6 (IL-6) and IL-8 [218]. Using the selective PPAR*β*/*δ* agonist GW501516, others also reported the critical role of PPAR*β*/*δ* in the suppression of IL-1**β**-induced *VCAM-1* and *E-selectin* expression in HUVECs [219]. At the molecular level, chromatin immunoprecipitation assays showed that ligand activation of PPAR*β*/*δ* in HUVECs switched the association of B cell lymphoma-6 (BCL-6), a transcription repressor and anti-inflammatory regulator, from PPAR*β*/*δ* to the vascular promoter of *VCAM-1* [219]. Such an unconventional ligand-dependent transcriptional pathway in which PPAR*β*/*δ* controls an inflammatory switch through its association and disassociation with the transcriptional repressor BCL-6 has been previously abundantly illustrated in macrophages foam cells [220]. 

Another way to limit the inflammatory response by the nuclear receptor PPAR*β*/*δ* in endothelial cells could partially involve its physical interaction with the Extracellular signal-Regulated Kinases (ERK). Notably, ERK was found to serve as an anti-inflammatory signal that suppresses expression of NF-*κ*B-dependent inflammatory genes by inhibiting IKK activity in endothelial cells [221]. Furthermore, ERK1, 2, and 5 enhance PPAR*β*/*δ* transcriptional activity in C2C12 murine myoblasts leading to a reduction in cytokine-mediated NF-*κ*B activation [67, 222]. Perhaps a similar molecular scenario could also take place in endothelial cells but it has not been documented yet. PPAR*β*/*δ* may therefore serve as a potent therapeutic target in inflammatory therapy.

### 8.3. PPAR*γ* and the Control of Endothelial Inflammation

The nuclear receptor *Ppar*γ** is also expressed in vessel wall tissue including endothelial cells, which are, together with macrophages and smooth muscle cells, key players in atherosclerosis development [223, 224]. A wealth of studies has previously shown that PPAR**γ** agonists can modulate the expression of many proinflammatory cytokines, chemokines, and adhesion molecules in endothelial cells [225, 226]. However, some PPAR**γ**-independent effects have been reported for certain PPAR**γ** agonists. Therefore, to circumvent the receptor-independent effect that individual PPAR**γ** agonists may display, a constitutively ligand-independent active mutant form of PPAR**γ**1 was delivered into human umbilical cord veins endothelial cells (HUVECs) [215]. Importantly, AP-1 and NF-*κ*B pathways were inhibited by the constitutively active form of PPAR**γ**1 in endothelial cells, leading to the prevention of endothelial activation, leucocyte recruitment, and synthesis of proinflammatory adhesion molecules. Definitive evidence that PPAR**γ** plays a functional role in regulating the inflammatory process* in situ* in endothelial cell comes with the establishment of LDL receptor-deficient mice deleted from *Ppar*γ** especially in endothelial cells [227]. Lack of *Ppar*γ** in primary endothelial cells leads to increased inflammation (as shown by the robust increased expression of *Tnf-*α**, *Mcp-1*, and *IL-1*β**) in vessel wall of mutant mice treated with LPS or challenged with high-cholesterol diet. In agreement with these findings, others have also recently reported that the genetic deletion of *Ppar*γ** in endothelium in mice was upregulating LPS signaling as the consequence of induction of NF-*κ*B activity [228].

Together, these data reinforce the notion that the pharmacological activation of PPAR**γ** is likely beneficial by limiting inflammation at the level of the endothelial cell as well.

**Figure 2:**
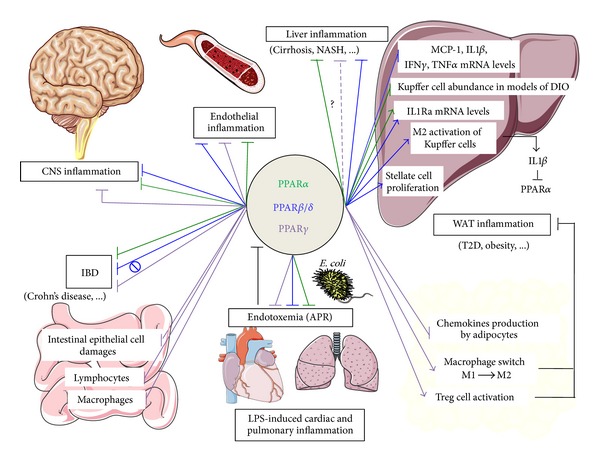
Representative illustration of PPAR main targets in inflammatory diseases. PPAR**α** mostly displays anti-inflammatory properties in the context of liver inflammation. Its reported liver targets are hepatocytes and Küppfer cells [131]. IL-1**β** produced by Küppfer cells potently suppresses *Ppar*α** expression and activity *via *NF-*κ*B–dependent inhibition of PPAR**α** promoter activity [160]. Besides downregulating gene expression of proinflammatory mediators such as *Mcp-1*, *Tnf-*α**, *Ifn-*γ**, *IL-1*β**, and PPAR**α** also directly controls expression of IL1-ra in liver [131, 163]. Küppfer cell activation is also dependent on PPAR*β*/*δ*, which also targets stellate cells and therefore prevents liver fibrosis [75, 138]. In addition, PPAR*β*/*δ* has well-established anti-inflammatory properties in diseases associated with CNS inflammation. In CNS, PPAR*β*/*δ* has also proven anti-inflammatory properties in neurons, glial cells, and astrocytes [200–202]. PPAR**γ** anti-inflammatory properties are mainly illustrated in T2D and IBD. PPAR**γ** serves as the molecular target of the insulin-sensitizing TZD drugs and plays a key role in T2D, adipogenesis and obesity. In WAT, mature adipocytes, Treg cells and macrophages have been identified as key cellular targets for PPAR**γ** [66, 75, 116, 117]. Macrophage-specific deletion of PPAR**γ** leads to specific reduction in alternatively activated macrophages (M2 state) in WAT leading to local inflammation [110]. Moreover, Treg-cell-specific deletion of *Ppar*γ** was shown to reduce the abundance of Treg cells in WAT resulting in the increase of WAT infiltration by proinflammatory macrophages (M1) and monocytes [116, 117]. In IBD, PPAR**γ** acts in intestinal epithelial cells, macrophages and lymphocytes [190–192]. Note that endotoxemia represses the mRNA expression level of *Ppars *(see black bar) [150–155]. Furthermore, multiple lines of evidence indicated that PPAR**γ** is very important in endothelial cells, because it inhibits the *in situ* production of proinflammatory molecules such as vascular adhesion molecule-1 (VCAM-1), intercellular adhesion molecule-1 (ICAM-1) and MCP-1 [215, 223–228]. Similar conclusions were also drawn for the PPAR**α** and PPAR*β*/*δ* isotypes [214, 216–220]. Finally, PPARs display protective effets against endotoxemia [166, 167, 169, 236]. NASH: nonalcoholic steatoHepatitis; T2D: type-2 diabetes; CNS: central nervous system; Treg cells: Foxp3^+^ CD4^+^ regulatory T cells; DIO: diet-induced-obesity; APR: acute phase response; green lines: action of PPAR**α**; blue lines: action of PPAR*β*/*δ*; purple lines: action of PPAR*γ*; ?: Some PPAR*γ*-independent effects of PPAR*γ* activators have been proposed [146, 147]; *∅*: pharmacological activation of PPAR*β*/*δ* did not protect against dextran sulfate sodium-induced colitis pointing towards a ligand-independent anti-inflammatory effect of PPAR*β*/*δ* [180].

In summary, all three PPARs isotypes display an anti-inflammatory role by inhibiting the production of inflammatory cytokines in a large set of syndromes and diseases (Figure 2).

## 9. Dairy Cattle and Mastitis: PPAR Modulators as Future Promising Treatment?

In livestock species in general, data describing the use of synthetic PPAR agonists are very limited. Considering the high-amino acid identities ranging from 95 to 98% for PPARs proteins in all species, one can think that bovine and porcine PPARs could also be targeted with existing synthetic PPAR agonists [229]. On the other hand, because only a minor overlap between the Wy-regulated genes from mouse and human primary hepatocytes was found and since PPRE are not fundamentally conserved along species, we have to admit that activation of PPARs does not necessarily activate the same array of genes in one species *versus* another [60, 61]. 

One of the most common diseases in dairy cattle in the world is mastitis, which can be defined as an inflammation of the mammary gland tissue, resulting from the introduction and multiplication of pathogenic microorganisms. Mastitis is one of the most important health problems and is very costly for the dairy industry [230]. While treatment is possible with long-acting antibiotics, farmers have to wait until drug residues have left the cow's system before milk from such cows becomes again marketable. Several main causative bacteria that include *Escherichia coli *are responsible for the induction of inflammation of the udder tissue in dairy cattle. We have illustrated above that PPAR**γ** activation, which typically results in the downregulation of inflammatory response, is suggested to be beneficial in inflammatory diseases not only in humans, but also in rats and pigs. We now question and discuss whether PPAR**γ** activation could mitigate immunological stress of livestock, such as mastitis. As its function is to recognize pathogens that have not been encountered before, the innate immune system is the first line of defense against intramammary infection by bacteria [231]. It is generally accepted that emigration from the blood vessel of neutrophils (also known as polymorphonuclear neutrophil leucocytes) into the infected tissue, where they will deliver antimicrobial agents, is a hallmark of bacterial infection. Given that during the APR, reduction of the neutrophil flux into the mammary gland is believed to promote the incidence of severe* Escherichia coli*-induced mastitis, it could perhaps be envisioned to counterbalance this effect by treatment with existing PPAR**γ** agonists [232]. Using two different mouse models of sepsis (cecal ligation and puncture as well as intraperitoneal injection of purified bacterial gram-negative LPS) it was rather shown that PPAR*γ* inactivation with the GW9662 compound significantly (i) reversed the suppression of chemotaxis observed following LPS administration and (ii) increased recruitment of PMNs in the peritoneal cavity of mice subjected to cecal ligation and puncture [233]. Therefore, PPAR**γ** displays two facets: once activated, it would dampen the massive production of proinflammatory cytokines in response to bacterial gram-negative LPS injection, by transrepressional mechanisms; at the same time, it would accentuate the suppression of chemotaxis further interfering with the recruitment of PMNs to the site of infection, two early key events for fighting against bacterial infection. Given that PPAR**γ** is also a pivotal NHR involved in adipocyte differentiation and fat mass, modulating its activity could also affect fat depots important for meat quality. Therefore, pharmacological interventions in dairy cattle based on the use of PPAR**γ** (anta) gonists may not offer an overall favorable therapeutic benefit, unless PPAR**γ** ability to control inflammation and interfere with PMN recruitment is disconnected in these pharmacological reagents. The recent generation of pigs, which display physiological and anatomical similarities with humans, in which one allele of the *Ppar*γ** gene has been disrupted could be partly informative concerning the real involvement of PPAR**γ** in the etiology of mastitis in livestocks [234].

Applications of PPAR**α** agonists could be of interest to decrease inflammation in the udder but since PPAR**α** signaling is decreased in bovine mammary tissue challenged with bacteria, and because fatty acid oxidation is under the dependence of PPAR**α** in the liver, the routine use of such molecules remains largely speculative [235]. 

## 10. Concluding Remarks

PPARs are lipid sensing transcription factors that were originally targeted in order to normalize metabolic issues. However, it also turned out that these NHRs were as well potently involved in switching off inflammation. Thanks to their respective and well conserved expression in numerous tissues amongst species, the prevalence of inflammatory diseases could be reduced by the use of a combination of different PPARs agonists. Quite surprisingly though, only a limited number of anti-inflammatory genes have been identified so far as direct and classical PPAR targets with a functional PPRE in genomic DNA, which could appear a bit puzzling at first glance. However, mechanisms involved in the anti-inflammatory properties of PPARs are broader than what might have been thought originally. Such properties are the reflect of a much elaborated transrepressional activity. The mechanisms behind this activity are currently being studied and remain more or less elusive at the moment. Therefore, it will be a major challenge for the future, in terms of therapeutic applications, to fully understand how these NHRs work and control inflammation. Compared to other anti-inflammatory strategies such as that involving glucocorticoids and its receptors, PPARs agonists may be responsible for limited drawbacks, yet their use also revealed controversial results in terms of efficacy and side effects.

## Figures and Tables

**Table 1:** Tissue distribution of the various PPARs in different species.

Specie	Tissue	Expression
	PPAR*α* (NR1C1)	

	Liver	++ [237]
	WAT	N.D.
Cow/cattle	GI tract	N.D.
	Brain	N.D.
	Spleen/thymus	N.D.

	Liver	++ [23]
Chicken	WAT	+ [82]
	Brain	++ [82]
	Spleen	+ [82]

	Liver	+++ [20, 61, 118, 162, 175]
	Primary hepatocytes	± to +++ [61, 134]
	HepG2 hepatoma cells	+ [54]
	HepaRG hepatoma cells	++ [134]
Human	WAT	+ [20, 118, 238]
	Isolated adipocytes	± [20]
	GI tract	++ [20, 118, 175, 239]
	Brain	+ [118, 175, 240]
	Monocytes	+ [241, 242]
	Dendritic cells	++ [241, 242]
	Kidney	++ [20, 118]
	Heart	+++ [118]

Pig	Liver	± [243]
	WAT	+ [243]

	Liver	+++ [61, 83, 244–247]
	Hepatocytes	++ [61]
	GI tract	++ [Nursa] [175]
Mouse/rat	Brain	+ [Nursa]
	Spleen/thymus	− [83]
	Macrophages (BMDM)	− [244]
	FAO hepatoma cells	++ [54]
	WAT	+ [62, 248]

	PPAR*β*/*δ* (NR1C2)	

	Liver	N.D.
	WAT	N.D.
Cow/cattle	GI tract	N.D.
	Brain	N.D.
	Spleen/thymus	N.D.

	Liver	N.D.
	WAT	N.D.
Chicken	GI tract	N.D.
	Brain	N.D.
	Spleen/thymus	N.D.

	Liver	± [20]
	HepG2 hepatoma cells	++ [54]
	WAT	± [20]
	Isolated adipocytes	± [20]
	Large intestine	+++ [20]
	Small intestine	+ [20]
Human	Colon mucosae (adult)	++ [239]
	Brain	N.D.
	Monocytes	++ [241]
	Macrophages	+++ [249]
	Dendritic cells	+ [241]
	Kidney	+ [20]
	Skeletal muscle	± [20]

	Liver	+ [250]
Pig	WAT	++ [250]
	Stomach	++ [250]
	Brain	++ [250]

	Liver	± [251]
Rabbit	GI tract	+ [251]
	Brain	++ [251]
	Spleen/thymus	± [251]

	Liver	+ to ++ [Nursa] [50, 83, 246, 247]
	FAO hepatoma cells	++ [54]
	WAT	+ [Nursa] [50]
Mouse/rat	GI tract	+++ [Nursa] [50, 175]
	Brain	+++ [Nursa] [50, 83, 193, 252]
	Macrophages (BMDM)	++ [244]
	Colon	++ [83]

	PPAR*γ* (NR1C3)	

	Liver	− [253]
	WAT	+++ [253, 254]
Cow/cattle	Spleen/thymus	++ [253]
	Small intestine	± [253]
	Mammary gland	[235]

	Liver	− [82]
Chicken	Spleen/thymus	+ [82]
	Brain	+ [255]
	WAT	+++ [255]

	Liver	+ [20, 256, 257]
	HepG2 hepatoma cells	+ [54]
	HepaRG cells	± [134]
	Primary hepatocytes	± [134]
	WAT	+++ [20, 54, 256, 257]
Human	Isolated adipocytes	+++ [20]
	Simpson-Golabi-Behmel Syndrome (SGBS) adipocytes	+++ [84]
	Large intestine	+++ [20]
	Small intestine	± [20]
	Brain	N.D.
	Monocytes	+++ [241]
	Dendritic cells	+++ [241]
	Kidney	+ [20]
	Skeletal muscle	± [20]

Pig	Liver	− [243]
	WAT	++ [243]

	Liver	− to + [251, 258]
	WAT	+++ [258]
Rabbit	GI tract	+++ [251]
	Brain	− [251]
	Spleen/thymus	++ [251]

	Liver	+ to – [Nursa] [83, 246, 247]
	Hepatocytes	+ [259]
	FAO hepatoma cells	− [54]
	WAT	+++ [Nursa] [83, 256, 260]
Mouse/rat	3T3-L1 adipocytes	+++ [84]
	GI tract	+ [Nursa] [83]
	Brain	+ [Nursa] [83, 261, 262]
	Spleen/thymus	++ [83]
	Macrophages (BMDM)	+++ [244]

Abbreviations: GI: gastrointestinal; WAT: white adipose tissue; N.D.: not determined. BMDM: bone marrow-derived macrophages.

Symbols: −: absent; ±: barely detectable; +: weak; ++: moderate; +++: high. the citation link for Nursa is http://www.nursa.org/10.1621/datasets.02001.
